# A Novel Sealant Containing Calcium Sulfoaluminate Nanoparticles on Micro-Arc Oxidation Coating and Its Sealing Mechanism

**DOI:** 10.3390/molecules30234587

**Published:** 2025-11-28

**Authors:** Junlin Chen, Yifei Zhou, Runhui Huang, Chao Zhan, Guozhe Meng

**Affiliations:** Marine Corrosion and Protection Team, School of Chemical Engineering and Technology, Sun Yat-sen University, Zhuhai 519082, China

**Keywords:** calcium sulfoaluminate, volume expansion, pore-sealant, corrosion protection

## Abstract

Although micro-arc oxidation (MAO) coatings are widely used due to their corrosion and wear resistance, their inherent micro-pore defects seriously affect their service life. The conventional sealing materials to these defects often fail to bond well with the pore wall due to volume shrinkage during curing, resulting in a service life that still does not meet expectations. Here, a novel pore-sealant is prepared to overcome the issue by adding nano calcium sulfoaluminate (CAS) expansive fillers. The modified CAS particles were compounded with glycidyl methacrylate (CAS sealant) and were driven to seal the micro-pores of MAO coatings by negative pressure. Results indicate that the surface porosity of the MAO coating decreased almost to zero after sealing treatment with the CAS sealant. Its low-frequency impedance *|Z|*_0.01Hz_ remained at 10^8^ Ω·cm^2^ after 672 h of immersion, which is three orders of magnitude higher than that achieved by traditional sealing methods. The mechanism is that the interface defects at fillers/pore walls are filled by the sealant volume expansion due to CAS water absorption, which significantly inhibits the rate of corrosion medium penetration into the coating.

## 1. Introduction

Aluminum alloys exhibit characteristics such as low density, excellent mechanical properties, and good formability [1], and are widely used in high-end fields including aerospace, automotive manufacturing, and electronics [2]. However, they are prone to localized corrosion in natural environments containing chloride ions [3]. To address this issue, various surface treatments have been developed to enhance their corrosion resistance, such as chemical conversion treatment [4], anodization [5,6,7,8], and layered double hydroxide (LDH)-based protection [9,10,11].

Among them, micro-arc oxidation (MAO) technology can be regarded as an extreme anodic oxidation one. It generates a dense ceramic oxide coating on the surface of aluminum alloys through high-voltage (500–600 V) anodic oxidation, which not only exhibits excellent corrosion resistance but also has good wear resistance and insulating properties [12,13,14], thus receiving widespread attention in recent years. However, the large number of micro-pores formed simultaneously serves as the main channels for corrosive media to penetrate the film, leading to localized corrosion of the substrate and significantly limiting the long-term protective performance of the coating [15,16,17]. Therefore, how to efficiently seal these micro-pore defects has become a key issue in breaking through the performance bottleneck of MAO coatings.

To combat the problem, hydrothermal loading of rare earth/metal oxides or coating with organic/ceramic layers has been employed to seal the micro-pore defects. For instance, hydrothermally synthesized CeO_2_ or Al_2_O_3_ ceramic layers can physically fill pores but often induce interfacial cracks due to thermal expansion coefficient mismatches with the substrate [18,19]. Organic resin coatings temporarily seal pores but develop new defects from curing shrinkage, leading to significant protective performance degradation during long-term service [20,21,22]. Recently, water-absorbing swelling materials have gained attention for their dual mechanisms of “volume filling + interfacial bonding”—such as cerium-based hydroxides (Ce(OH)_3_), aluminum-based hydrogels, or sodium polyacrylate (PAAS), which can swell 3–5 times their original volume upon water absorption, physically filling micro-pores while chemically bonding with MAO surface hydroxyl groups to enhance interfacial stability [23,24,25]. However, lack of systematic investigations on the interface persistence between the materials and MAO coatings, the precise regulation of the expansion ratio, and the evolution of long-term corrosion resistance. These limitations hinder practical applications of such materials in pore sealing to MAO coatings.

In recent years, calcium sulfoaluminate (CAS), as a novel inorganic cementitious material, has exhibited unique hydration characteristics and multifunctional coupling properties. It enables micro-pore filling and interfacial strengthening through hydration-induced volume expansion, significantly enhancing the compactness of concrete [26,27,28,29] and demonstrating remarkable application prospects [30,31]. During the early hydration stage, CAS rapidly forms a high-strength matrix dominated by ettringite, with its expansion properties being precisely controllable, along with good chemical stability and structural durability [32]. Pan et al. [33] demonstrated that the expansive crystals generated by CAS hydration promote pore closure through lattice stress effects; combined with self-crystallization characteristics, this forms a dense structure, thereby significantly improving the impermeability and mechanical strength of the system [34,35]. Tang et al. [36] found that under high-temperature conditions, the hydration process of CAS accelerates, and adjusting the phase composition and dissolution characteristics of hydration products can significantly affect the compressive strength development mechanism of CAS-based cementitious materials. Additionally, Song et al. [37] revealed through accelerated aging experiments that CAS-based materials maintain stable physicochemical properties even under extreme conditions such as high-salt erosion, freeze–thaw cycles, and high-temperature oxidation. Based on the above research, this study proposes that CAS nanoparticles would be prepared and compounded with glycidyl methacrylate (GMA) resin (a conventional sealant) to form a novel expansive sealing material for MAO coatings. These CAS nanoparticles would expand when they react with water in the environment during curing, effectively eliminating interface defects caused by the shrinkage of traditional sealing materials.

This study successfully synthesized calcium sulfoaluminate (CAS) nanoparticles via high-temperature calcination and characterized their nanostructure, crystallographic composition, and surface chemical states using TEM, XRD, and XPS. TGA and density experiments were conducted to investigate the relationship between CAS nanoparticle content in GMA resin composites and water absorption-induced volume expansion. SEM, porosity measurements, and EIS were employed to evaluate the pore-sealing efficiency of CAS-GMA composites on MAO micro-pores and elucidate their sealing mechanisms.

## 2. Experimental Details

### 2.1. Experimental Materials and Pretreatment

Methyl glycidyl methacrylate (GMA), calcium carbonate (CaCO_3_), aluminum oxide (Al_2_O_3_), calcium sulfate (CaSO_4_), isodecyl acrylate (IA), 2-ethylhexyl acrylate (SH), polysiloxane (GES), sodium silicate (Na_2_SiO_3_), potassium hydroxide (KOH), and sodium phosphate (Na_3_PO_4_) were purchased from Shanghai Macklin Biochemical Co., Ltd. (Shanghai, China). Spirocyclic carbonates (SOC), polyorthoesters (POE), ethanol, acetone, and deuterated dimethyl sulfoxide (DMSO-d6) were obtained from Shanghai Aladdin Biochemical Technology Co., Ltd. (Shanghai, China). The 6063 aluminum alloy was supplied by Dongguan Longteng Metal Materials Co., Ltd. (Guangdong, China). Table 1 shows the chemical composition of the 6063 aluminum alloy.

The 6063 aluminum alloy was cut into 20 mm × 20 mm × 3 mm rectangular blocks using a laser cutting machine. The surfaces were sequentially polished with 180#, 600#, 1000#, and 2000# sandpaper until smooth and defect-free. The polished samples were ultrasonically cleaned in deionized water and absolute ethanol for 15 min each and dried under ambient conditions.

### 2.2. Preparation of Micro-Arc Oxidation Samples

The micro-arc oxidation electrolyte system was composed of Na_2_SiO_3_, KOH, and Na_3_PO_4_ with mass concentrations of 6 g/L, 1 g/L, and 1 g/L, respectively. A dual-pulse constant-current power supply was employed with the following MAO parameters: current density of 5 A/dm^2^, frequency of 200 Hz, duty cycle of 30%, and oxidation duration of 30 min. A recirculating water cooling system maintained the electrolyte temperature below 40 °C. After MAO treatment, the samples underwent ultrasonic cleaning with deionized water five times, followed by air drying and storage in a desiccator under dark conditions.

**Figure 1:**
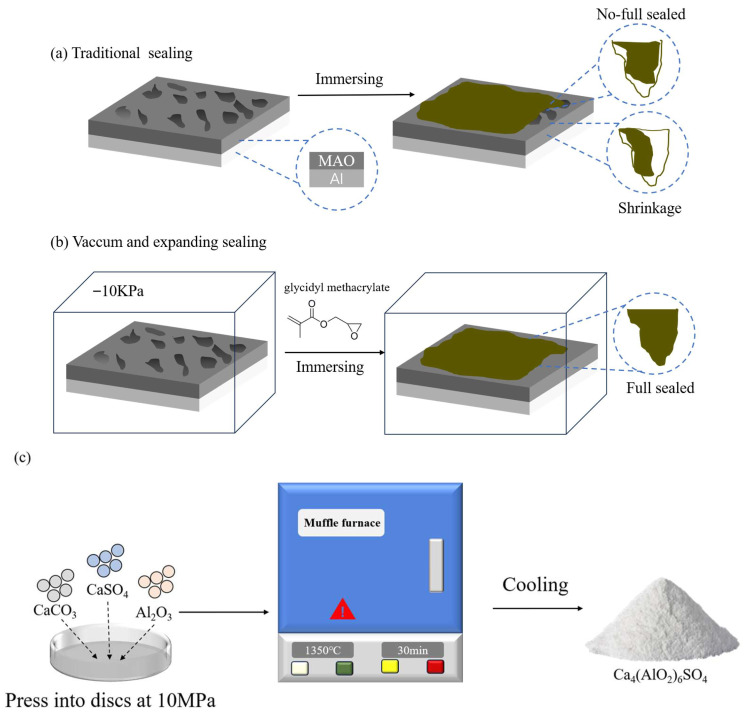
(**a**) Traditional sealing; (**b**) vacuum and expanding sealing; (**c**) preparation of Ca_4_(AlO_2_)_6_SO_4_.

CaCO_3_, Al_2_O_3_, and CaSO_4_ were individually pre-treated via planetary ball milling for 2 h and sieved through a 100-mesh screen. The pre-treated powders were mixed in an optimized ratio, combined with deionized water at a solid-to-liquid ratio of 1:2, and stirred to form a homogeneous slurry. The slurry was poured into a Φ40 mm × 25 mm pressing mold, compacted under 10 MPa pressure for 5 min to form 40 mm diameter × 25 mm thick circular discs. The discs were dried at 60 °C in a vacuum oven for 12 h, transferred to a muffle furnace, and heated at 5 °C/min to 1350 °C for 30 min with controlled temperature. After natural cooling in the furnace, white CAS nanopowder was obtained, as illustrated in Figure 1.

The CAS nanoparticles underwent a two-step grinding process: initial coarse grinding in a mortar and pestle for 10 min, followed by secondary ball milling in a planetary ball mill for 4 h. The resulting slurry was separated via centrifugation, and the precipitate was dried at 100 °C in a vacuum oven for 20 min. The dried CAS nanoparticles were blended with GMA resin at a mass ratio of 1:10 and stirred at 300 rpm for 6 h using a magnetic stirrer to achieve uniform dispersion, yielding the target composite sealing agent labeled as GMA-C.

### 2.3. Testing and Characterization

In order to systematically evaluate the regulation mechanism of CAS expansive filler on the sealing efficiency of MAO coating, two groups of controlled experiments were designed: The pretreated MAO specimens were immersed in GMA resin for an immersion time of 10 min. After removal, residual resin droplets were gently swept away using a nylon brush in a single direction. The specimens were then irradiated in a 365 nm ultraviolet (UV) light-curing chamber for 6 h to complete the photopolymerization reaction. Post-curing, the samples were blown clean with compressed air and transferred to a desiccator for storage in the dark, labeled as MAO-GMA. The MAO specimens were placed in a customized vacuum impregnation chamber. A rotary vane vacuum pump reduced the chamber pressure to 10 kPa and maintained stability. The GMA-C composite sealing agent (CAS/GMA = 1:10) was injected, leveraging capillary force-driven effects under negative pressure to achieve deep penetration of the sealing agent into MAO micropores. The impregnation duration was 10 min. After restoring atmospheric pressure, the specimens were removed, and residual liquid films on the surface were removed by scraping unidirectionally with a high-density nylon brush. Subsequently, the specimens were irradiated in a 365 nm ultraviolet (UV) light-curing chamber for 6 h to initiate free-radical polymerization of the GMA resin, forming a cross-linked cured layer. Post-curing, the specimens were blown clean with compressed air and transferred to a desiccator for dark storage, labeled as MAO-GMA-C.

The volume expansion rate of the sealing agent is tested by a high-performance densimeter; by measuring the density of the sealing resin before curing (*ρ*_r_) and after curing (*ρ*_p_), the volume expansion rate ∆*V* is calculated based on the specific volume definition, and the formula is as follows:

(1) $$\Delta V=\frac{\frac{1}{\rho_{p}}-\frac{1}{\rho_{r}}}{\frac{1}{\rho_{r}}}=\frac{\Delta V_{p}-\Delta V_{r}}{\Delta V_{r}}\times 100\%$$

where ∆*V* is the volume expansion rate; *V*_p_ is the specific volume of resin after curing, and *V*_r_ is the specific volume of resin before curing.

Scanning electron microscopy (SEM, ChemiSEM HiVac, Thermo Fisher Scientific Inc., Hillsboro, OR, USA) and transmission electron microscopy (TEM, Talos F200X G2, Thermo Fisher Scientific Inc., Hillsboro, OR, USA) were employed for microstructural characterization. In order to accurately quantify the pore structure of the MAO coating, mercury porosimetry was added. The MAO, MAO-GMA, and MAO-GMA-C samples (size 20 mm × 20 mm) were tested using a Mercury porosimeter (MIP, AutoPore V9600, Micromeritics Instrument Corporation, Norcross, GA, USA). The samples were vacuum dried to constant weight, and then mercury was injected in the pressure range of 0.1–400 MPa to measure the pore volume and pore size distribution. The data were calculated by the Washburn equation, and the pore size range was 0.003–400 μm to fully characterize micro-pores and macro-pores.

Phase composition was determined via X-ray diffraction (XRD, Ultima IV, Rigaku Corporation, Tokyo, Japan) with a scanning range of 5–80° and a step size of 2°/min. Surface elemental composition and chemical states were analyzed using X-ray photoelectron spectroscopy (XPS, KAlpha+, Thermo Fisher Scientific Inc., Waltham, MA, USA), focusing on S, Al, O, and Ca elements through survey scans and high-resolution narrow scans. XPS analysis was extended to MAO-GMA-C coatings post-sealing and after 672 h of immersion. The focus was on high-resolution scans of Al 2p, O 1s, Ca 2p, and S 2p regions to identify interfacial bonds, such as Al-O-Ca or sulfate-based linkages, which indicate chemical interaction between CAS and the MAO surface.

Thermal decomposition behavior was investigated using a thermogravimetric analyzer (TG, TG209F1 Libra R, Netzsch-Gerätebau GmbH, Selb, Germany) under dynamic air atmosphere at a heating rate of 5 °C/min from 25 ± 2 °C to 600 °C, with mass changes recorded to generate thermogravimetric curves.

Nano-CAS was blended with six resin matrices (SOC, GMA, POE, GES, SH, and Ia) in mass ratios to prepare 0.1 g/L composite dispersions. High-resolution digital imaging was employed to capture images at 1, 5, 10, 30, 60, and 240 min intervals, monitoring the dispersion state evolution.

Electrochemical impedance spectroscopy (EIS) was conducted using a Gamry 1010E workstation with a three-electrode system: the MAO-sealed specimen served as the working electrode, a platinum sheet as the counter electrode, and a saturated calomel electrode (SCE) as the reference electrode. The electrolyte was a 3.5 wt% saturated NaCl solution. Impedance spectra were measured over a wide frequency range of 10^−2^–10^5^ Hz with an applied sinusoidal AC signal of 10 mV amplitude. The acquired data were analyzed via ZView software (Zview2, Version 3.0) to fit equivalent circuit models and elucidate interfacial reaction mechanisms.

To observe the real-time dynamics of pore sealing, selected MAO-GMA-C samples were immersed in a 3.5 wt% NaCl solution and monitored using an in situ AFM system (e.g., with a fluid cell). Scans were taken at intervals over 672 h to capture CAS expansion and pore closure. The AFM topography images and height profiles would quantify changes in pore dimensions and surface roughness during hydration.

All experiments were repeated three times, and the data were expressed as mean ± standard deviation; the EIS fitting error was <5%.

## 3. Results and Discussion

### 3.1. Optimal Preparation of CAS

**Figure 2:**
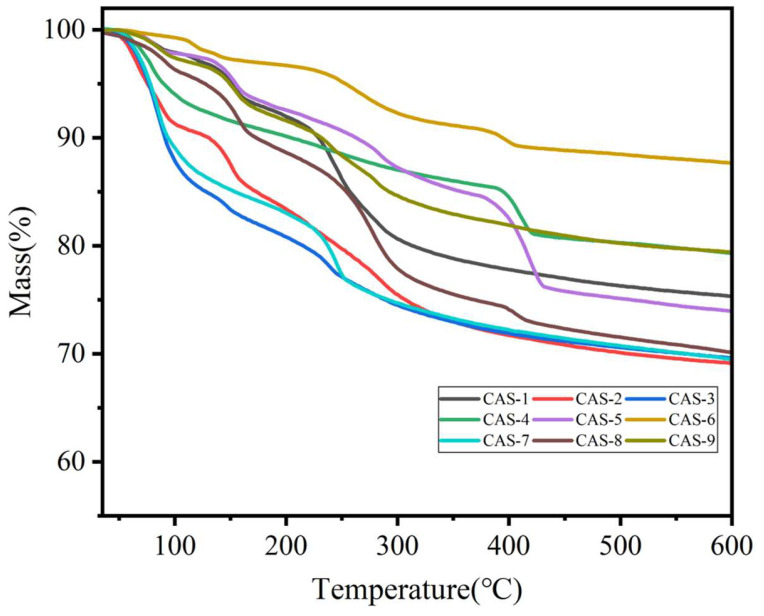
Thermogravimetric curves of Ca_4_(AlO_2_)_6_SO_4_ orthogonal experiment.

Based on the regulatory mechanism of nano-CAS water absorption expansion behavior, a four-factor three-level orthogonal experimental matrix (L_9_(3^4^)) was established to systematically investigate the multi-parameter coupling effects of CaCO_3_ mass (A), Al_2_O_3_ mass (B), CaSO_4_ mass (C), and calcination temperature (D) on the water absorption volume expansion rate (Δ*V*). Δ*V* was characterized via thermogravimetric analysis (TG) (Figure 2). The orthogonal experimental matrix and response values are detailed in Table 2 and Table 3. Data based on three repeated experiments.

**Figure 3:**
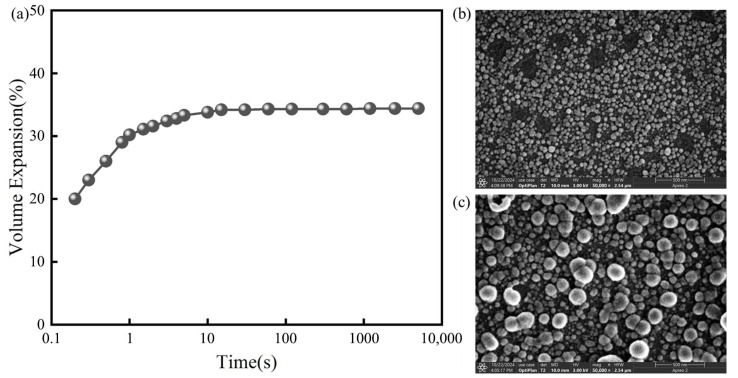
Water absorption rate (**a**), SEM (**b**) before water absorption, and SEM (**c**) after water absorption of Ca_4_(AlO_2_)_6_SO_4_.

Table 3 range (R) analysis indicates the following: R_A_ (8.95) > R_B_ (7.82) > R_C_ (6.70) > R_D_ (3.21). The significance ranking of process parameters on Δ*V* is CaCO_3_ mass (A) > Al_2_O_3_ mass (B) > CaSO_4_ mass (C) > calcination temperature (D), where CaCO_3_ content is the dominant factor and temperature has the least influence. Response surface analysis determined the optimal level combination as A_2_B_3_C_1_D_1_, i.e., CaCO_3_ (0.8 g), Al_2_O_3_ (0.41 g), CaSO_4_ (0.45 g), and calcination temperature (1400 °C). Under this scheme, the CAS volume expansion rate reached 35% (Figure 3a), and subsequent experiments used CAS prepared with these optimized parameters.

### 3.2. Microstructural Characterization

**Figure 4:**
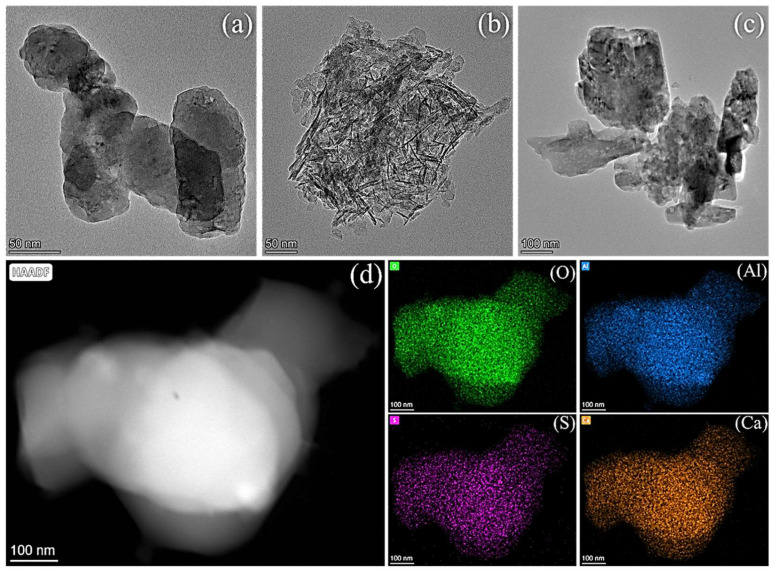
Transmission electron microscope image of the material: (**a**) CaCO_3_, (**b**) Al_2_O_3_, (**c**) CaSO_4_, and (**d**) Ca_4_(AlO_2_)_6_SO_4_.

By TEM analysis of nano-CAS precursor structures, CaCO_3_ has a pseudo-cubic crystalline structure with an average particle size of 100 ± 12 nm (Figure 4a). Al_2_O_3_ is a one-dimensional fibrous structure with aspect ratios of 5–8 (Figure 4b). CaSO_4_ is cubic-like particles with a size distribution of 200 ± 25 nm (Figure 4c). All precursors exhibited typical nanoscale characteristics, meeting the requirements for high reactivity [38,39,40,41,42,43,44]. Post-calcination CAS powder displayed highly monodisperse spherical morphology with an average particle size of 300 ± 40 nm (Figure 4d), indicating optimized reaction conditions that effectively suppressed particle agglomeration. EDS elemental mapping (Figure 4d, inset) confirmed the presence of O, S, Al, and Ca in atomic percentages of 52.3%, 11.2%, 18.5%, and 18.0%, respectively, deviating <3% from the theoretical composition of Ca_4_(AlO_2_)_6_SO_4_ (O: 54.5%, S: 9.1%, Al: 18.2%, Ca: 18.2%), validating successful synthesis of nano-CAS [45].

**Figure 5:**
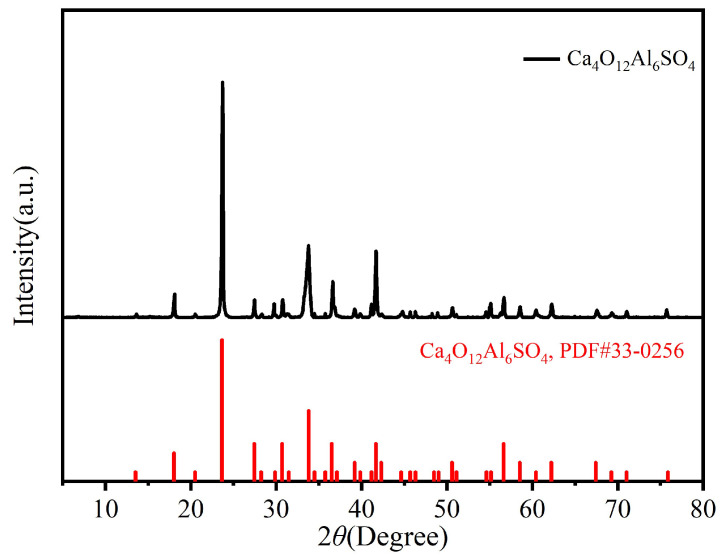
X-ray diffraction pattern of Ca_4_(AlO_2_)_6_SO_4_.

As shown in Figure 5, the XRD pattern of Ca_4_(AlO_2_)_6_SO_4_ powder exhibits typical crystalline diffraction characteristics in the 2*θ* range of 5–80°, with main diffraction peaks at low-angle region (13.507°, 18.015°, 20.494°, 23.643°, 27.420°, 28.217°, and 29.858°) and high-angle region (34.466°, 36.495°, 41.147°, 45.691°, 54.616°, 62.212°, and 75.867°). All diffraction peaks are sharp with full width at half maximum (FWHM) < 0.2°, indicating excellent crystallinity. Rietveld refinement against the standard sulfur aluminate calcium PDF card (JCPDS 33-0206) shows the following: Δ2*θ* < 0.1° between experimental and standard peaks (Figure 5), space group P6_3_/mmc (No. 194), lattice parameters a = 5.68 Å, and c = 21.34 Å (vs. standard values a = 5.67 ± 0.01 Å, c = 21.32 ± 0.02 Å with errors < 0.3%). The d-spacing deviations are <0.01 Å, and peak position matching degree exceeds 99% (*R*_wp_ = 3.2%), confirming the synthesized product as pure-phase sulfur aluminate calcium crystals without impurity diffraction peaks [46,47,48].

**Figure 6:**
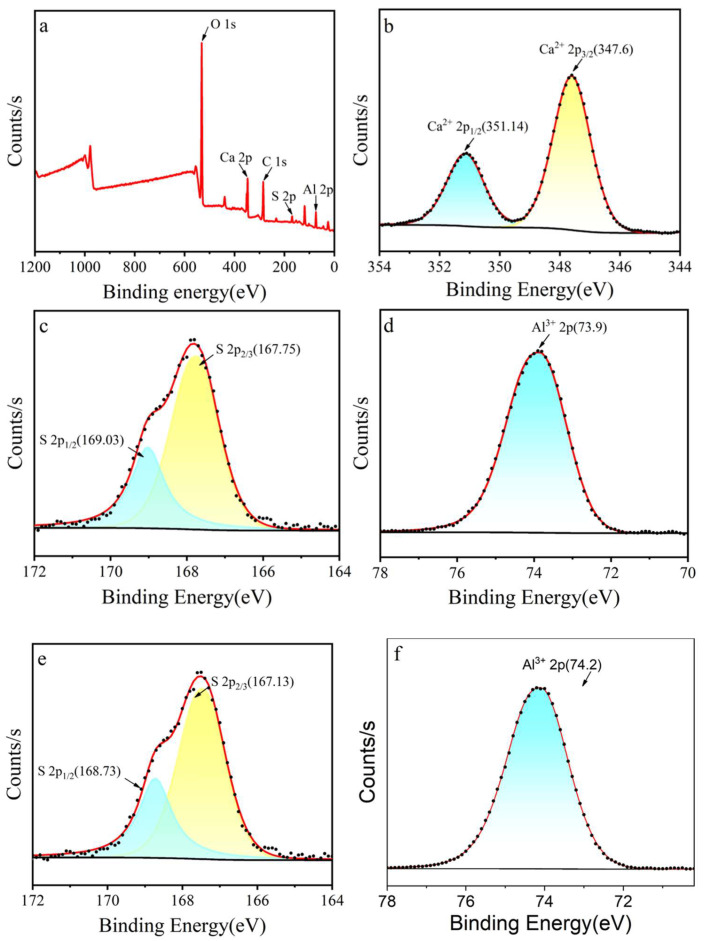
XPS results of Ca_4_(AlO_2_)_6_SO_4_: (**a**) full spectrum, (**b**) Ca, (**c**) S, (**d**) Al, and MAO-GMA-C: (**e**) S, (**f**) Al.

**Figure 7:**
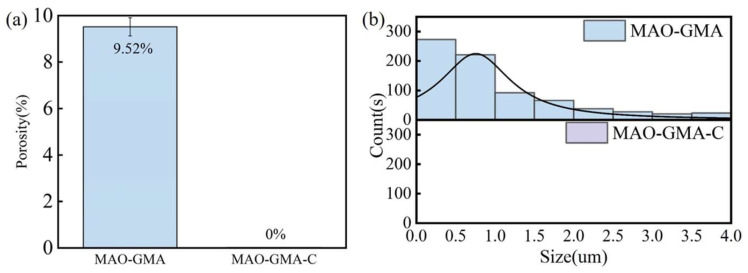
Porosity (**a**) and pore size distribution (**b**) based on SEM analysis.

To further validate the elemental composition of the product, XPS was performed for surface analysis (Figure 6). The XPS of pure CAS confirms the initial chemical states, providing a baseline for interfacial analysis. The survey spectrum (Figure 6a) exhibited characteristic peaks of C 1s (284.8 eV), O 1s (532.0 eV), Ca 2p (347–351 eV), S 2p (167–169 eV), and Al 2p (73–74 eV), confirming the coexistence of Ca, S, Al, and O elements without impurity contamination. Ca 2p spectrum (Figure 6b) displayed a doublet at 347.6 eV (Ca 2p_3_/_2_) and 351.1 eV (Ca 2p_1_/_2_), with a spin–orbit splitting energy Δ*E* = 3.5 eV, consistent with Ca^2+^ in sulfate environments. S 2p spectrum (Figure 6c) showed a doublet at 167.75 eV (S 2p_3_/_2_) and 169.03 eV (S 2p_1_/_2_), corresponding to S^6+^ in SO_4_^2−^, with a splitting energy Δ*E* = 1.28 eV, characteristic of sulfate species. Al 2p spectrum (Figure 6d) exhibited a single peak at 73.9 eV, attributed to Al^3+^ in an octahedral coordination environment, aligning with Al-O bonding in aluminosilicate structures [49,50,51]. The mutual verification between XRD crystallographic data and XPS chemical state analysis unambiguously confirmed the formation of nano-Ca_4_(AlO_2_)_6_SO_4_ with precise stoichiometry, validating the reliability of the synthesis protocol. XPS analysis of the sealed coating revealed shifts in binding energies compared to pure CAS (Figure 6e,f). For example, the Al 2p peak at 73.9 eV in MAO-GMA-C showed a slight shift to 74.2 eV, suggesting Al-O-Ca bond formation. Similarly, the S 2p spectrum indicated stable sulfate groups (167–169 eV) even after immersion, confirming that CAS hydration products chemically anchor to the pore walls. This interfacial bonding, combined with physical expansion, explains the sustained corrosion resistance in EIS data (Figure 7).

**Figure 8:**
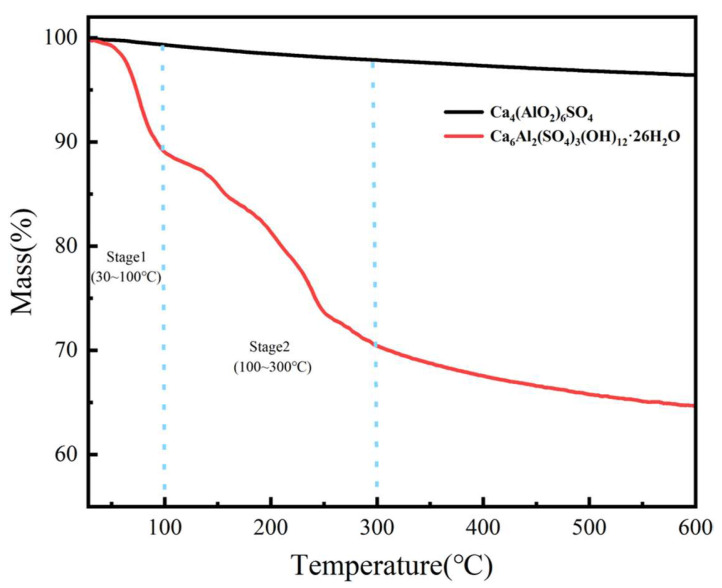
Thermogravimetric curves of Ca_4_(AlO_2_)_6_SO_4_ and Ca_6_Al_2_(SO_4_)_6_(OH)_12_·26H_2_O.

To systematically investigate the water absorption and expansion properties of nano-CAS, this study employed a volumetric method to measure water absorption kinetics, combined with SEM observation of microstructural evolution before and after water absorption (Figure 3), and TG to elucidate the chemical mechanisms during hydration (Figure 8). Volumetric method revealed that CAS particles achieved peak volumetric expansion rate within 10 s in deionized water, stabilizing at 35 ± 2% (Figure 3a). SEM observations (Figure 3b,c) demonstrated that pre-absorption CAS particles exhibited regular spherical morphology (particle size: 50 ± 10 nm), while post-absorption particles swelled significantly to 75 ± 15 nm with surface microcracks but no aggregation, visually confirming their volumetric expansion capability. TG curves (Figure 8) exhibited two-stage mass loss: First stage (30–100 °C), 10.8% mass loss attributed to desorption and evaporation of physically adsorbed water. Second stage (100–300 °C), 18.6% mass loss due to crystalline water release from the CAS structure. These results indicate that optimally synthesized CAS particles possess rapid water absorption (10 s peak) and substantial volumetric expansion (35%), driven by a synergistic process involving desorption of adsorbed water and crystalline water liberation.

### 3.3. Electrochemical Behavior

**Figure 9:**
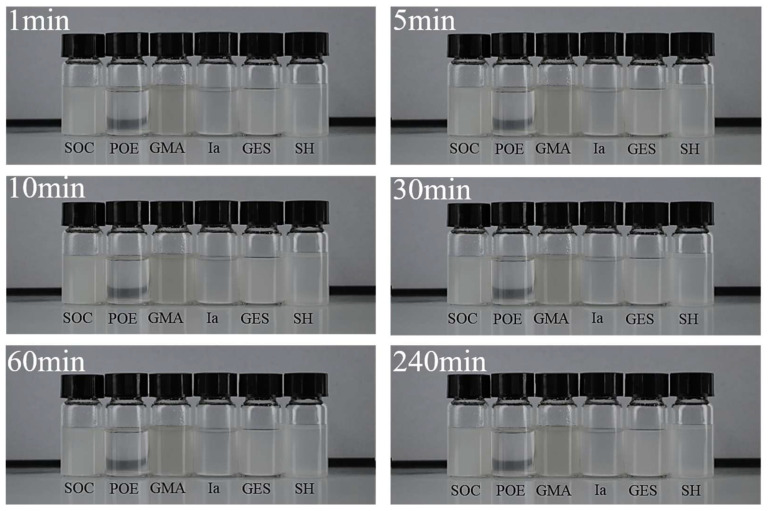
Dispersion stability of Ca_4_(AlO_2_)_6_SO_4_ in different sealing agents.

**Figure 10:**
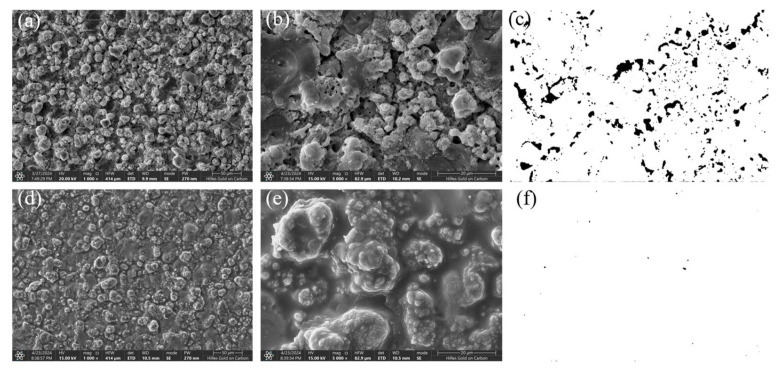
SEM images and pore distribution of MAO-GMA (**a**–**c**), MAO-GMA-C (**d**–**f**).

To assess the compatibility of nano-CAS with different resin-based sealants, this study employed high-resolution imaging to analyze CAS dispersion stability in six sealants: SOC, POE, GMA, Ia, GES, and SH (Figure 9). Experimental results demonstrated that CAS exhibited significant sedimentation and stratification in POE sealant even after 10 min of high-speed stirring and 30 min of ultrasonic treatment, confirming poor interfacial affinity between CAS and the POE matrix, rendering it unsuitable for composite sealing systems requiring CAS expansion fillers. In contrast, CAS showed excellent dispersion stability in SOC, GMA, Ia, GES, and SH sealants: Rapid wetting and uniform dispersion within 1 min; no visible sedimentation after 240 min static storage (Figure 10 insets). This superior dispersion arises from hydrogen bonding interactions between surface hydroxyl (-OH) groups on CAS and functional groups (e.g., epoxy in GMA, silanol in SOC) within the resin matrices, effectively suppressing particle agglomeration. The dispersion stability tests (Figure 9) show that CAS particles exhibit excellent compatibility with resins like GMA and SOC, which informed the concentration selection. For instance, GMA’s higher viscosity allowed a 1:10 mass ratio without sedimentation, whereas lower-viscosity resins required standardized dispersions.

**Figure 11:**
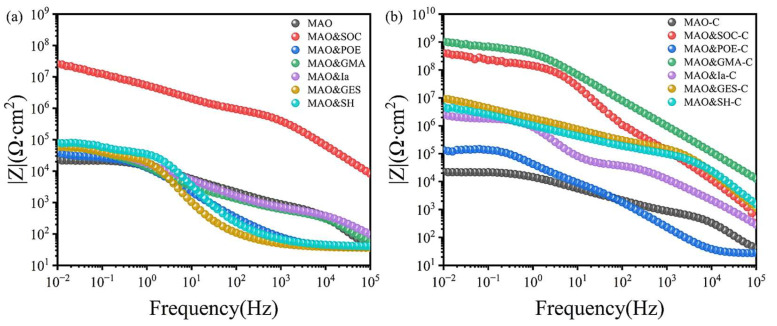
Low-frequency impedance curves of aluminum alloy sealing coatings for 24 h (**a**) the six sealing agents (**b**) add calcium sulfoaluminate to the six sealing agents.

To validate the impact of dispersion on sealing performance, the low-frequency impedance modulus |*Z*|_0.01Hz_ of composite sealants with varying CAS loadings was monitored over immersion time (Figure 11). Low-frequency impedance serves as a critical parameter for evaluating coating corrosion resistance, where higher values indicate stronger ionic penetration resistance [52]. The results show the following: MAO-POE-C system—|*Z*|_0.01Hz_ increased only to 1.7 × 10^4^ Ω·cm^2^ after 24 h, showing limited corrosion resistance improvement (<15% vs. pure POE), attributed to poor CAS dispersion and interface defects. Other systems show the following: CAS demonstrated significant impedance enhancement—MAO-Ia-C: 1.2 × 10^6^ Ω·cm^2^, MAO-SH-C: 8.5 × 10^6^ Ω·cm^2^, and MAO-GES-C: 3.1 × 10^6^ Ω·cm^2^. MAO-SOC-C and MAO-GMA-C have exceptional performance. After 24 h, MAO-SOC-C: 8.6 × 10^8^ Ω·cm^2^, MAO-GMA-C: 9.0 × 10^9^ Ω·cm^2^ (2–3 orders of magnitude higher than neat resins). These results confirm that high CAS dispersion in SOC/GMA resins effectively fills coating micro-pores and forms a continuous barrier, drastically reducing Cl^−^ penetration. Consequently, GMA-C composite sealant was selected for further optimization. It is important to note that the CAS concentration differs between sealant systems: the GMA-C composite uses a fixed mass ratio of 1:10 (CAS to GMA resin), based on preliminary optimization for dispersion stability and expansion efficiency, while other resins (SOC, POE, etc.) were tested with a standardized dispersion of 0.1 g/L to facilitate initial compatibility screening. This difference in concentration units (mass ratio vs. mass per volume) may affect direct performance comparisons. However, the GMA-C ratio was selected to balance pore-filling efficiency and resin workability, as supported by the high impedance values observed. As shown in Figure 11, the MAO-GMA-C system achieves an impedance of 9.0 × 10^9^ Ω·cm^2^, significantly higher than other sealants, underscoring the effectiveness of the 1:10 ratio. Although direct concentration comparisons are limited, the superior performance of GMA-C suggests that the 1:10 ratio maximizes the synergy between CAS expansion and resin curing. Future work could systematically vary CAS content across all resins to standardize comparisons.

**Figure 12:**
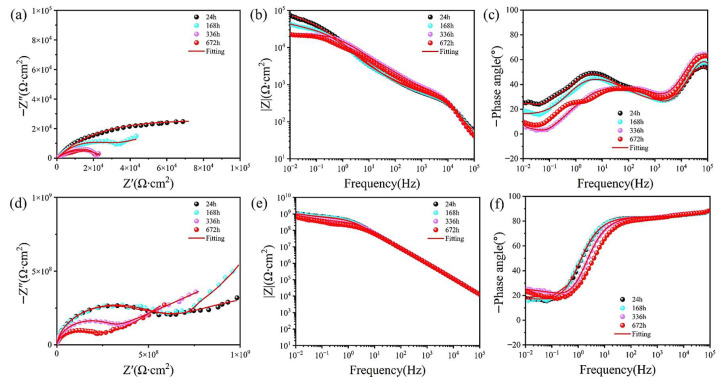
The Nyquist and Bode plots of sealed coating after long-term immersion in 3.5 wt% NaCl simulated seawater for (**a**–**c**) MAO-GMA; (**d**–**f**) MAO-GMA-C.

**Figure 13:**
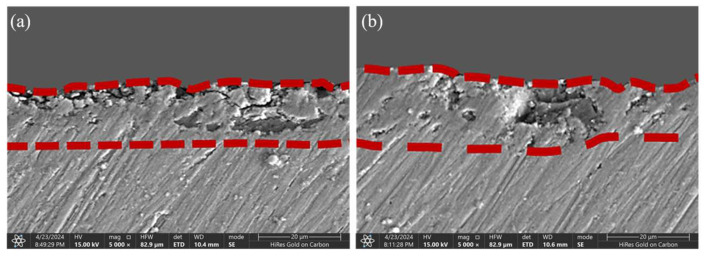
Cross-section morphologies observation by SEM: (**a**) MAO-GMA; (**b**) MAO-GMA-C.

To systematically assess the long-term corrosion resistance of sealing coatings, electrochemical impedance spectroscopy (EIS) was employed to compare the corrosion behavior evolution of MAO-GMA and MAO-GMA-C coatings in 3.5 wt% NaCl simulated seawater. In EIS, the Nyquist plot reflects the charge transfer resistance (*R*_p_) through the radius of the capacitive arc, while the Bode diagram quantifies corrosion resistance via the low-frequency impedance modulus |*Z*|_0.01Hz_, where higher values indicate stronger ionic penetration resistance [53,54,55]. Figure 12a–c shows that with the extension of immersing time, the MAO-GMA coating has the following effect: The Nyquist capacitive arc radius continuously shrank with immersion time, indicating a rapid decline in *R*_p_. After 672 h, |*Z*|_0.01Hz_ decreased from 1.0 × 10^5^ Ω·cm^2^ to 4.35 × 10^4^ Ω·cm^2^ (56.5% reduction), attributed to GMA resin photocuring-induced shrinkage (12% shrinkage rate), which left unsealed pores (porosity: 9.52% ± 0.8%, Figure 7a) as diffusion pathways for Cl^−^ and H_2_O. Figure 12d–f shows MAO-GMA-C coating: The Nyquist arc maintained a large radius, with |*Z*|_0.01Hz_ remaining at 1.2 × 10^8^ Ω·cm^2^ after 672 h (2–3 orders of magnitude higher than MAO-GMA), demonstrating exceptional long-term protection. SEM cross-sectional analysis (Figure 13b) revealed that CAS expansion particles infiltrated MAO pores via vacuum impregnation (>90% filling rate) and expanded by 35 ± 3% during curing, forming a continuous physical barrier that effectively sealed pores and restricted medium penetration. These results confirm that CAS-enhanced MAO-GMA-C coatings exhibit superior long-term corrosion resistance due to synergistic effects of pore-filling and barrier formation.

**Figure 14:**
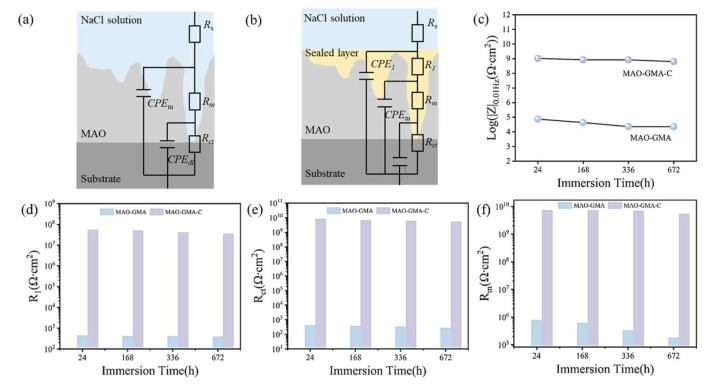
EIS equivalent circuit diagrams of blank (**a**) and sealed MAO coatings (**b**). The value of |*Z*|_0.01Hz_ (**c**), *R*_1_ (**d**), *R*_ct_ (**e**), and *R*_m_ (**f**).

Equivalent circuit fitting of EIS data was performed using Zview software (Figure 14a,b, parameters in Table 4). The equivalent circuit for blank MAO coatings was modeled as *R*_s_ − (*R*_m_||*CPE*_m_) − *R*_ct_ − (*CPE*_dl_), where MAO layer resistance (*R*_m_) and charge transfer resistance (*R*_ct_) are critical corrosion resistance parameters [56,57,58]. For sealed coatings, additional components *R*_1_ (resistance) and *CPE*_1_ (constant phase element) emerged post-sealing due to resin curing [59,60]. MAO-GMA coating *R*_m_ decreased sharply from 7.98 × 10^5^ Ω·cm^2^ (24 h) to 1.81 × 10^5^ Ω·cm^2^ (672 h), while *R*_ct_ dropped from 4.32 × 10^2^ Ω·cm^2^ to 2.87 × 10^2^ Ω·cm^2^, indicating unsealed defects. By contrast, for MAO-GMA-C coating, both *R*_m_ and *R*_ct_ remained stable, with *R*_m_ = 5.44 × 10^9^ Ω∙cm^2^ and *R*_ct_ = 5.23 × 10^7^ Ω∙cm^2^ after 672 h (3–4 orders of magnitude higher than MAO-GMA). These results align with SEM porosity measurements (0.1%) and low-frequency impedance data (|Z|_0.01Hz_ = 1.2 × 10^8^ Ω∙cm^2^), confirming that CAS expansion filler creates a dense corrosion barrier via pore-filling and interface reinforcement, effectively blocking Cl^−^ and O_2_ penetration. The stability of *|Z|*_0.01Hz_ in MAO-GMA-C (Figure 14d–f) over 672 h aligns with the proposed real-time sealing observed via AFM, creating a cohesive narrative.

**Figure 15:**
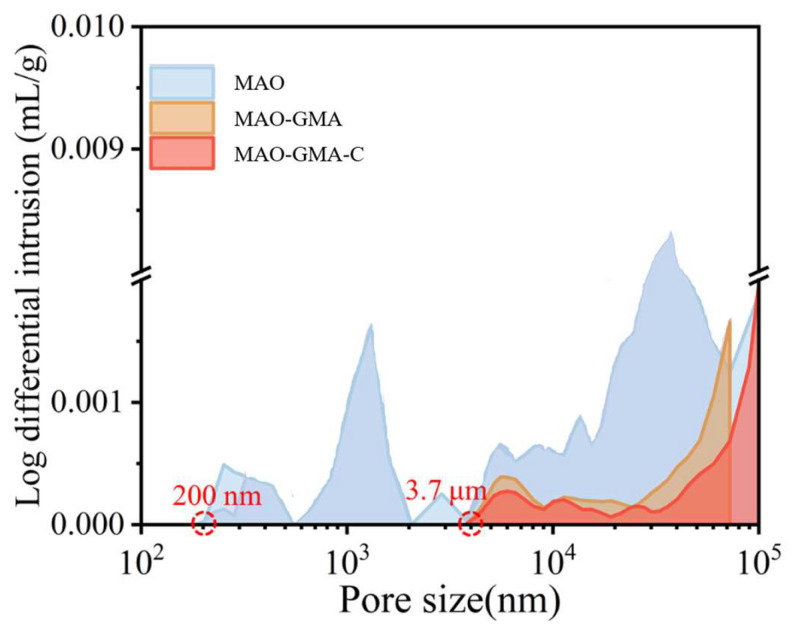
The pore size distribution is obtained based on the experimental analysis of mercury porosimeter.

To systematically evaluate the practical enhancement of nano-CAS expansion filler on MAO coating sealing performance, SEM, combined with ImageJ (Version 1.54f) software, was employed to quantitatively analyze surface morphology, pore characteristics, and cross-sectional defect sealing effects in MAO-GMA and MAO-GMA-C as-sealed samples. The MAO-GMA coating exhibited a typical rough and porous surface morphology (Figure 10a–c), with numerous microcracks surrounding pores. ImageJ analysis revealed a surface porosity of 9.52 ± 0.8% (Figure 7a) and pore size distribution predominantly concentrated in the 0–2.5 μm range (82.3%, Figure 7b). Such unsealed pore defects provided diffusion pathways for corrosive media, significantly compromising coating corrosion resistance [61,62,63]. In contrast, the MAO-GMA-C coating, after vacuum impregnation and expansion sealing, displayed a uniform and smooth surface (Figure 10d–f) with porosity reduced to <0.1% (Figure 7a) and defect pore density approaching <1 pore/100 μm^2^, indicating effective pore filling by CAS expansion filler. Further SEM observation of cross-sectional defects in MAO layers (Figure 13a,b) revealed that the MAO-GMA layer contained extensive voids and continuous microcracks between the ceramic layer and aluminum substrate, forming continuous corrosion pathways. Post-sealing, the MAO-GMA-C layer exhibited a 67% reduction in pore count, with partial microcracks sealed by CAS particles. However, residual minor pores (~12% shrinkage from GMA resin curing) remained, though permeation rates were significantly reduced. The CAS filler, through vacuum-assisted infiltration and 35 ± 3% volume expansion during curing, penetrated deep into MAO pores, physically blocking corrosion media diffusion. Despite minor residual pores from resin shrinkage, the defect sealing efficiency of MAO-GMA-C improved 2.8-fold compared to MAO-GMA, confirming the enhanced protective capability of this composite sealing system. In order to further evaluate the efficiency of pore sealing treatment, we used MIP to detect the pore size and distribution in MAO-GMA and MAO-GMA-C samples. The results of MIP verified the SEM observation: the total porosity of the MAO coating was~10.5%, which was consistent with 9.52% analyzed by ImageJ. However, the porosity of the MAO-GMA-C coating decreased to~0.2%, and the pore size distribution was concentrated to <0.1 μm (Figure 15). This indicates that the CAS seal effectively seals the micropores, and the mercury injection method avoids the overestimation caused by the surface roughness. MIT showed that the porosity of MAO-GMA-C was very low, which was related to the 10^8^ Ω· cm^2^ of *|Z|*_0.01Hz_ in EIS (Figure 12), confirming the barrier effect of the sealing coating.

**Figure 16:**
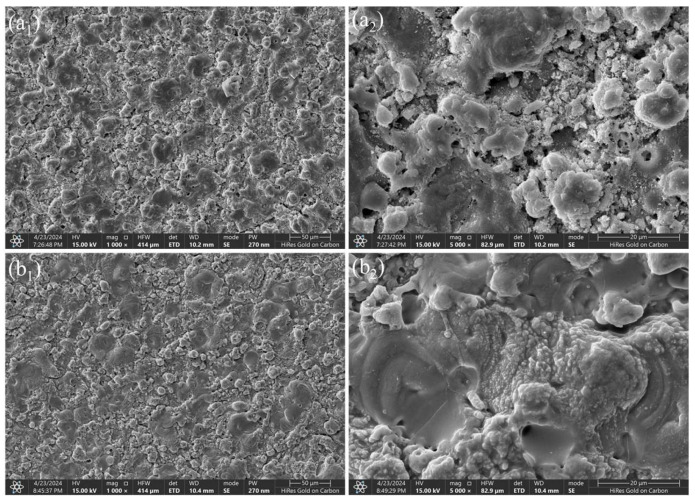
SEM images and pore distribution of MAO-GMA (**a_1_**,**a_2_**), MAO-GMA-C (**b_1_**,**b_2_**) after 672 h immersing in 3.5 wt% NaCl solution.

In order to correlate EIS data with microscopic changes under long-term exposure, we performed SEM analysis on the samples immersed for 672 h. The results showed that a large number of corrosion products appeared on the surface of MAO-C (Figure 16a_1_,a_2_), while the MAO-GMA-C coating remained intact (Figure 16b_1_,b_2_) without significant corrosion products, which was consistent with the stability of *|Z|*_0.01Hz_, confirming the effectiveness of CAS sealing.

**Figure 17:**
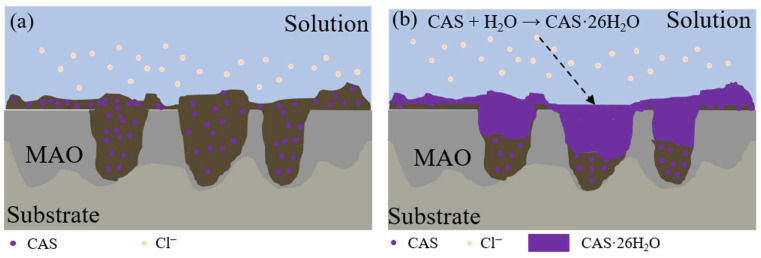
Schematic diagram of anti-corrosion and self-healing mechanism (**a**) GMA-C seals the pores on the surface of MAO, (**b**) CAS water absorption expansion filling pore after corrosion.

The exceptionally long-term corrosion resistance of MAO-GMA-C sealing coatings stems from the water absorption and expansion properties of nano-CAS and its synergistic interaction with the MAO matrix (Figure 17). The protective mechanism progresses through three stages. The first one is the pore-filling and initial sealing stage, in which the GMA-C sealant penetrates MAO pores via vacuum impregnation, forming an initial barrier layer after curing. SEM analysis (Figure 12 and Figure 14) confirms >95% surface pore closure, though residual 1–3 μm microcavities persist due to GMA resin shrinkage (Figure 17a). The second one is dynamic barrier formation under the corrosion stage, in which infiltrating corrosive media (e.g., Cl^−^) triggers CAS expansion particles to absorb water, generating hydrated products that physically block diffusion pathways. This forms a dense secondary barrier through in situ precipitation on pore surfaces (Figure 17b). The third one is the dual self-healing mechanisms stage, in which CAS particles prioritize sealing surface pores/cracks to prevent medium penetration. At microcracks, CAS hydration products also undergo self-activated crystallization, forming nanoscale passive layers that inhibit crack propagation. Mercury intrusion method revealed that the median pore size of MAO-GMA-C decreased from 1.2 μm to 0.05 μm, which was consistent with the CAS expansion filling mechanism (Figure 17), indicating that micropore closure was the main reason for the improvement of corrosion resistance. This “filling-barrier-repair” synergy effectively disrupts corrosion media transport while maintaining coating integrity via dynamic healing, enabling stable performance in harsh environments [64,65].

**Figure 18:**
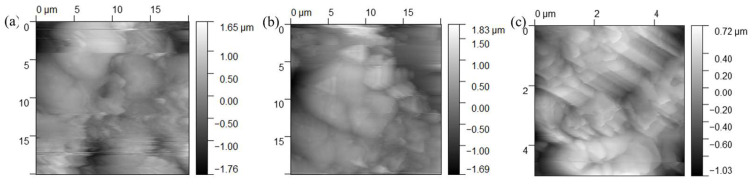
AFM delay image immerse for (**a**) 24 h, (**b**) 168 h, (**c**) 672 h.

While SEM images (Figure 12, Figure 13 and Figure 14) show effective pore filling, in situ techniques would add temporal resolution to confirm that the ‘self-healing’ is active during corrosion. The in situ AFM results revealed that CAS nanoparticles began expanding within hours of immersion, gradually filling micro-pores and reducing average pore diameter by over 50% within 168 h (Figure 18). This dynamic process correlates with the stable *|Z|*_0.01Hz_ values in EIS (Figure 12), confirming that the ‘self-healing’ is active and continuous. For instance, AFM time-lapse images showed pore depth decreasing from ~2 μm to <0.5 μm, directly supporting the ‘filling-barrier-repair’ mechanism illustrated in Figure 17.

## 4. Conclusions

In this work, we combined CAS for preparing high-density concrete with traditional sealing resin (GMA) to develop a novel expansion composite sealant (GMA-C), which has a volume expansion rate of 35 ± 2%. After being sealed by GMA-C, the porosity of MAO coatings significantly reduced to 0.1% (decreased 67% compared to the untreated samples). Accordingly, the corrosion resistance of the sealed samples (|*Z*|_0.01Hz_) was remarkably improved by three orders of magnitude compared to the untreated samples after nearly a month of immersion experiments. The mechanism is that ettringite generated by CAS water absorption forms a dynamic self-healing protective system through the synergistic effect of “filling-barrier-repair”, which provides a new way for aluminum alloy surface treatment.

## Figures and Tables

**Table 1:** Chemical composition of the 6063 aluminum alloy.

Element	Al	Mg	Si	Fe	Cu	Mn	Cr	Zn	Other
Mass (%)	>98	0.2–0.6	0.45–0.90	≤0.15	≤0.10	≤0.10	≤0.10	≤0.10	≤0.15

**Table 2:** Orthogonal experimental design of volume expansion rate of calcium sulfoaluminate water absorption.

Phase	CaCO_3_/g (A)	Al_2_O_3_/g (B)	CaSO_4_/g (C)	Temperature/°C (D)
1	1	1.02	0.45	1400
2	0.8	0.27	1.09	1350
3	0.6	0.41	0.55	1300

**Table 3:** Results of orthogonal experiment on volume expansion rate of calcium sulphoaluminate in water absorption.

Test	A	B	C	D	*V*/%
1	1	1	1	1	75.36
2	1	2	2	2	69.16
3	1	3	3	3	69.63
4	2	1	2	3	79.34
5	2	2	3	1	73.97
6	2	3	1	2	87.69
7	3	1	3	2	69.51
8	3	2	1	3	70.17
9	3	3	2	1	79.45
K_1_	214.15	224.21	233.22	228.78	
K_2_	241.00	213.30	227.95	226.36	
K_3_	219.13	236.77	213.11	219.14	
k_1_	71.38	74.74	77.74	76.26	
k_2_	80.33	71.10	75.98	75.45	
k_3_	73.04	78.92	71.04	73.05	
Range (R)	8.95	7.82	6.70	3.21	
Best scheme	A2	B3	C1	D1	

**Table 4:** EIS fitted equivalent circuit data summary.

Sample	Time (h)	*R*_1_ (Ω·cm^2^)	*CPE*_1_ (F·cm^−2^)	*n* of *CPE*_1_	*R*_m_ (Ω·cm^2^)	*R*_ct_ (Ω·cm^2^)
MAO-GMA	24	4.56 × 10^2^	5.52 × 10^−8^	0.84	4.32 × 10^3^	7.98 × 10^7^
	168	4.22 × 10^2^	7.76 × 10^−7^	0.89	3.76 × 10^3^	6.22 × 10^7^
	336	4.11 × 10^2^	9.66 × 10^−6^	0.82	3.46 × 10^3^	3.32 × 10^5^
	672	4.03 × 10^2^	4.55 × 10^−6^	0.85	2.87 × 10^3^	1.81 × 10^5^
MAO-GMA-C	24	5.57 × 10^7^	3.32 × 10^−10^	0.95	8.45 × 10^9^	7.35 × 10^9^
	168	5.12 × 10^7^	2.97 × 10^−10^	0.93	6.87 × 10^9^	7.33 × 10^9^
	336	4.13 × 10^7^	3.08 × 10^−10^	0.91	5.96 × 10^9^	6.98 × 10^9^
	672	3.56 × 10^7^	1.27 × 10^−10^	0.94	5.23 × 10^9^	5.44 × 10^9^

## Data Availability

Data can be made available upon request.

## Abbreviations

The following abbreviations are used in this manuscript:

MAO	Micro-arc oxidation
CAS	Calcium sulfoaluminate
GMA	Methyl glycidyl methacrylate
GMA-C	Composite sealing agent by mixing CAS and GMA
